# Towards Mass Spectrometry-Based Chemical Exposome: Current Approaches, Challenges, and Future Directions

**DOI:** 10.3390/toxics7030041

**Published:** 2019-08-18

**Authors:** Jingchuan Xue, Yunjia Lai, Chih-Wei Liu, Hongyu Ru

**Affiliations:** 1 Center for Environmental Health and Susceptibility, University of North Carolina at Chapel Hill, Chapel Hill, NC 27599, USA; 2 Department of Population Health and Pathobiology, North Carolina State University, Raleigh, NC 27607, USA

**Keywords:** chemical exposome, biomonitoring, environmental monitoring, mass spectrometry, disease, bioinformatics

## Abstract

The proposal of the “exposome” concept represents a shift of the research paradigm in studying exposure-disease relationships from an isolated and partial way to a systematic and agnostic approach. Nevertheless, exposome implementation is facing a variety of challenges including measurement techniques and data analysis. Here we focus on the chemical exposome, which refers to the mixtures of chemical pollutants people are exposed to from embryo onwards. We review the current chemical exposome measurement approaches with a focus on those based on the mass spectrometry. We further explore the strategies in implementing the concept of chemical exposome and discuss the available chemical exposome studies. Early progresses in the chemical exposome research are outlined, and major challenges are highlighted. In conclusion, efforts towards chemical exposome have only uncovered the tip of the iceberg, and further advancement in measurement techniques, computational tools, high-throughput data analysis, and standardization may allow more exciting discoveries concerning the role of exposome in human health and disease.

## 1. Introduction

Studies have demonstrated that environmental factors play an equal or even more significant role in the pathogenesis of human chronic diseases compared with other risk factors such as genetic variants [1,2,3,4]. Environmental factors can induce changes in the human genome, transcriptome, epigenome, proteome, and metabolome. In 2005, the concept of exposome was first proposed by Christopher Wild to account for the unexplained risk factors underlying human diseases [5]. The “exposome” concept has shaped the thinking of scientists when studying environment-disease associations by switching the research paradigm from a single exposure-disease model to an agonistic analysis of environmental influences on human health [6,7,8]. Many research areas are benefiting from this mindset shift, including environmental epidemiology, health risk assessment, biomonitoring and environmental monitoring, and mechanistic biology. A considerable amount of published commentaries and reviews have highlighted the potential benefits of the characterization and integration of exposome in future studies [9,10,11,12,13,14,15,16,17].

The definition of exposome has been evolving ever since its birth. Wild originally defined it as “the totality of environmental exposures from birth onwards” [5]. Later, he redefined the scope of exposome to include three broad categories of non-genetic exposure: internal (e.g., metabolism, gut microbiome, inflammation), specific external (e.g., environmental pollutants, diet, occupation), and general external (e.g., socioeconomic status, education, and climate) [18]. In 2014, Miller and Jones expanded the Wild definition to include the measures of biological responses to these exposures [19].

Exposome studies aim to accomplish two critical goals: (1) to measure the cumulative exposures throughout the entire life of humans and (2) to evaluate the associations or causal relationships between these exposures and any biological changes. Depending on the specific types of exposure, exposome can be measured through a wide array of techniques including remote sensors, questionnaires, geography information systems, biomonitoring and environmental monitoring, and metabolomics [13,20,21]. As a comprehensive review of exposome is extremely large in scope, in this paper, we focus on the entire chemical exposures from embryo onwards, “chemical exposome”. 

There is still a long way to go to completely characterize the human chemical exposome. However, efforts are being made to capture a critical portion of it [22,23,24]. The aim of this paper is to undertake a systematic review of published evidences regarding the measurement techniques for the chemical exposome and available studies linking chemical exposome to human health. We further discuss the challenges confronted in implementing the exposome concept. Furthermore, we propose potential solutions to address the scientific and technological barriers to advancing exposome research.

## 2. Exposome Measurement Approaches 

Measuring the exposome has been a challenging task because of its complex and dynamic nature. To capture the full spectrum of exposures of interest in an individual’s life, many approaches have either been newly developed or transferred from other fields. For ease of description, we group the available approaches into three categories: chemical approaches (directly measure the exposures or early biomarkers using chemistry techniques), biological approaches (measure the biological changes induced by exposures using molecular biology techniques), and other approaches (those techniques which do not belong to either chemical or biological approaches, such as a personal wearable device). This paper focuses on the available mass spectrometry-based measurement techniques, but briefly discusses other approaches.

### 2.1. Chemical Approaches

Mass spectrometry based analytical techniques are widely used in biomonitoring and environmental monitoring studies and have become the predominant chemical approach in characterizing chemical exposome [9,25]. Mass spectrometry, coupled with a separation technique such as liquid or gas chromatography, has been the most popular method used in the direct measurement of chemicals (xenobiotics, their metabolites, and the early biomarkers) in biological or environmental samples due to the superiority in sensitivity, specificity, and dynamic range [9,25]. Low-resolution mass spectrometry has been traditionally used in targeted analytical methods to measure one or several classes of known chemicals in the sample [26,27,28]. High-resolution mass spectrometry-based biomonitoring techniques are considered very promising tools in achieving a more complete understanding of the biological significance of exposome [8,9,25].

#### 2.1.1. Low-Resolution Mass Spectrometry

Low-resolution mass spectrometers (LRMS), such as the triple quadrupole mass analyzer (QqQ), is only able to achieve *m*/*z* accuracy at unit level (~1 amu mass window) and is not capable of distinguishing compounds with very similar molecular mass. Also, LRMS has shown a low sensitivity in full scan mode. Both restrictions limit the ability of LRMS to detect unknowns. However, the selected reaction monitoring (SRM) mode of LRMS allows the quantitative analyses of a list of target precursor-product ion transitions with high sensitivity and broad linear dynamic range. To ensure the identification of a compound with LRMS, the retention time, at least two transitions (two product ions of the precursor ion), and their ratio of intensity are normally required [29]. False positive identifications are possible when only one transition is used [30]. 

Traditional biological measurement (targeted analysis) of exposures heavily relies on LRMS, which measures target xenobiotic compounds or their metabolites in the biological samples. This analytical platform typically provides validated and reliable quantification for analytes even at trace levels. This is of critical value for exposome studies because concentrations of xenobiotics are normally low in biospecimens [22]. Biomonitoring data in current databases or health surveys are primarily obtained via this approach because of its availability and maturity. For instance, the National Biomonitoring Program (NBP), launched by the Centers for Disease Control and Prevention (CDC) in the U.S., is routinely measuring approximately 300 chemicals which are known to be toxic to human beings [31]. New chemicals are added to the list when sufficient evidence supports the toxicological relevance and occurrence in the human body. 

However, LRMS based targeted analysis has several limitations when it comes to exposome: (1) the inability to cover a wide range of chemicals in a single run; (2) the high possibility of missing the compounds which are at high levels in the biospecimens but not in the target analyte list; (3) the limited availability of commercial standards to quantify the target analytes in the samples; (4) the potential of false positives due to certain precursor ions only generating one fragment ion; and (5) the limiting of detection of certain chemicals in analysis due to matrix interferences.

Efforts have been taken to capture the exposome with this targeted approach. To increase the coverage, scientists seek to measure as many chemicals as possible in one sample through multiple runs. In a recent study, 128 persistent and non-persistent endocrine disruptors belonging to 13 chemical classes were measured with both liquid chromatography-tandem mass spectrometry (LC-MS/MS) and gas chromatography mass spectrometry (GC-MS) [32]. Although this approach is costly, laborious, and needs a large volume of biological samples, it can provide validated information about the quantities of a relatively large number of chemicals in the human body. Further, with the technical advances in mass analyzers, the latest generation of QqQ instruments allows a notable increase in the number of transitions acquired within the same run. For instance, based on the triggered multiple reaction monitoring (tMRM) function in Agilent 6400 series QqQ instruments, a multiresidue LC-MS/MS method was established with a coverage of about 450 globally important pesticides within 10 min [33]. Several multi-target screening LC-MS/MS methods have also been established to simultaneously measure up to 700 drugs with the hybrid triple quadrupole linear ion trap technology (QTrap) [34,35]. 

#### 2.1.2. High-Resolution Mass Spectrometry

High-resolution mass spectrometers (HRMS) overcome the drawbacks of LRMS by providing high-quality mass resolution, exact molecular mass, and high sensitivity in full scan mode [36,37], which allows less strict requirements for the chromatography separation and improves the capability to detect low abundance chemicals in complex samples. Common HRMS typically possess mass-resolving power > 10, 000 (R, defined at full width at half maximum, FWHM), including time of flight (TOF), Fourier Transform Orbitrap (FT-Orbitrap), and Fourier transform-ion cyclotron resonance-mass spectrometer (FT-ICR-MS). FT-ICR-MS is costly and has limited availability and user feasibility, followed by FT-Orbitrap and TOF instruments. Currently, a vast majority of biomonitoring and environmental monitoring studies are based on TOF instruments, followed by FT-Orbitrap, with FT-ICR-MS platform generally not used in this field [25]. The pros and cons of TOF and FT-Orbitrap have been reviewed in multiple review articles [36,37,38]. Firstly, the FT-Orbitrap typically possesses higher mass-resolving power and mass accuracy than TOF instruments [38]. Mass-resolving power is highly dependent on the molecular composition, molecular mass, and scan speed [38]. Q Exactive Hybrid Quadrupole-Orbitrap is one of the commonly used FT-Orbitrap instruments and its resolution can be up to 1000 K at *m*/*z* of 200. Secondly, FT-Orbitrap instruments under full scan mode can achieve similar sensitivity down to a femtogram as QqQ instruments. As one of the newest versions of TOF instruments, Agilent 6550 iFunnel Q-TOF LC/MS can achieve a mass resolving power greater than 25K at 322 *m*/*z*. However, the sensitivity of TOF instruments under full scan mode is compromised with 1–2 orders of magnitude lower than that of QqQ operating in SRM mode [38]. This limits the capability of TOF instruments to measure chemicals at trace levels in samples. Thirdly, FT-Orbitrap instruments show an inverse relationship between scanning speed and mass resolution, while TOF instruments can maintain a rapid scanning rate regardless of the resolving power. Thus, the FT-Orbitrap based instrumental method needs to be well optimized to achieve a compromise between required mass resolution and adequate chromatography to adequately serve the research purpose [39]. Further, it needs to be noted that the application of an automatic gain control (AGC) in Orbitrap instruments is employed to prevent the overfilling of C-trap, but it also can affect the detection capability of trace compounds in complex matrices by reducing the absolute number of analyte ions that enter the ion trap [40]. The multiplexing feature of Q-Orbitrap instruments can help overcome this problem and achieve a higher sensitivity and an extended intrascan dynamic range for low-abundance chemicals in the complex matrices [40]. 

HRMS is typically combined with other mass analyzers to form hybrid instruments in real applications, including quadrupole (Q)-TOF, ion trap (IT)-TOF, Q Exactive, LTQ-Orbitrap, and Orbitrap Fusion Lumos Tribrid MS [36]. These hybrid mass analyzers can offer additional advantages such as increased sensitivity and fragments information for structural elucidation. In general, three analytical approaches have been developed with these hybrid mass spectrometers for exposome studies. 

***Targeted analysis***. In HRMS based targeted analysis, standards and information of target analytes are available. There are several advantages of HRMS based targeted analysis over LRMS based analysis. Firstly, the confidence in compound identification increases significantly by using exact mass, especially for those chemicals that have only one transition and non-specific transitions (e.g., neutral loss of H_2_O or CO_2_, which are also common for matrix interferences). It has been reported that an excellent selectivity regardless of the matrices could be achieved for most compounds with an R = 30,000, by which the target analytes could be distinguished from interferences of the same nominal *m*/*z* [38]. The separation of isobars generally requires higher mass resolving power, for instance, the isobars glutamine and lysine are separated with a multiple-reflection TOF MS in excess of 70 K after a flight time of 0.2 ms [41]. Except for the transitions and retention times, isotopic pattern and monoisotopic mass of analytes can also be used for the identification. Secondly, a higher coverage of analytes can be achieved in a single run. Many hybrid mass analyzers, like Q-TOF and Q Exactive, offer data-dependent MS/MS acquisitions. For example, if a compound in a target ion list is detected in the full scan mode, an MS/MS analysis will be triggered accordingly. This allows for full scan product ion spectra recording within the same run for a significant number of compounds at superior sensitivity than the limit of most LRMS. The limitation of TOF instruments based targeted analysis lies in the lower sensitivity and linear dynamic range compared with the QqQ [42,43]. However, many studies have achieved better sensitivity employing full scan or full MS-data dependent MS^2^ mode with FT-Orbitrap instruments compared with QqQ in targeted analysis [44,45,46]. 

***Suspect screening (biased non-targeted analysis)***. This approach is used when two conditions are met: compound-specific information (e.g., molecular formula, chemical structure, and physicochemical properties) of the suspects are known and the reference standards for the suspects are not available. M+1 and M+2 isotopes of precursor ion are critical in identifying the chemical formula and MS/MS spectral information is critical in elucidating the chemical structure. Using either Q-TOFMS or Orbitrap instruments, many studies have employed this analytical approach in successfully identifying suspect transformation products/metabolites of parent compounds in environmental media, such as natural waters and wastewater [47,48]. Recently this suspect screening technique was used for human samples. One study used GC-TOF/MS platform and found the presence of pentachlorobiphenyl in child brain tissue [49]. Another study used LC-QTOF/MS platform for the identification of new environmental organic acids (EOAs) in maternal plasma and discovered 65 suspect EOAs including benzophenone-1 and bisphenol S [50]. The available information of the compounds can help facilitate the acquisition and identification of suspects. Data-dependent acquisition mode is normally used in suspect screening, in which the sample analysis starts with full scan MS acquisition and switches to MS/MS mode when an analyte of interest appears in the run and is recognized by the data software based on pre-determined criteria [25]. Triggers normally used in the data-dependent acquisition mode have been reviewed in details, including ion intensity, accurate mass inclusion, isotope pattern, pseudo-neutral loss, and mass defect criteria [25]. When a suspect ion is found in the samples, chemical structure and physicochemical property-derived information can be used in the identification and confirmation of the compounds [47]. 

***Unknown screening (unbiased non-targeted analysis)***. When starting without any prior information about the compounds to be detected, an unknown screening approach is employed. Unknown screening theoretically enables the measurement of an unlimited number of compounds in the sample, making it a promising technique for the full characterization of the exposome. Workflows in analyzing HRMS data in non-targeted metabolomics studies have been reviewed elsewhere [25,51,52,53]. In exposome studies, the workflow can be borrowed from metabolomics with minor modifications, as detailed below.

(i) grouping all the features (ions or peaks) from the same compound based on the full scan MS chromatogram and determining monoisotopic or neutral molecular mass. An enormous number of ions are present in the chromatogram, but not every ion represents an individual compound. One compound may form different adducts (e.g., protonated and deprotonated ions, [M + Na]^+^, and [M + NH_4_]^+^), neutral losses (e.g., [M + H-H_2_O]^+^), isotopes (e.g., M+1 and M+2 isotope of precursor ion), and even in-source fragments. A variety of strategies have been proposed to group peaks including comparing expected theoretical distances between known ion adduct masses with experimental distances. One recent study suggested to extract MS pseudospectra based on peak shape and peak abundance based on the assumption that all the peaks with different *m*/*z* ratios from the same compounds ideally have a similar peak shape and strong linear relation in relative abundance across samples [51].
(ii) acquiring a list of potential chemical candidates by searching the monoisotopic mass or the molecular formula assigned against the databases. It has been reported that monoisotopic mass-based searching resulted in a higher percentage of chemicals in the number one rank position than chemical formula-based searching [54]. Available chemical substance databases such as PubChem and ChemSpider have been reviewed [52]. Recently, a few more databases have been developed for search including CompTox Chemistry Dashboard, Exposome-Explorer, and Toxic Exposome Database (T3DB) [55,56,57].
(iii) ranking the candidate list based on other information of the unknown chemical, including MS/MS spectral information, retention time, biochemical pathway and environmental chemistry knowledge. Fragments information can differentiate molecules with the same neutral mass in most cases. There are databases with experimental or in silico MS/MS spectral information available for reference, such as the Human Metabolome Database (HMDB) and METLIN. Currently available mass spectral databases have been reviewed elsewhere [52,58]. Retention time information can be obtained through quantitative structure-retention relationships (QSRR) models when reference standards are not available [59,60]. Biochemical pathway and environmental chemistry knowledge can also be used to narrow down putative identified compounds. In metabolomics, many bioinformatics tools are using biochemical pathways to filter and rank lists of candidates such as XCMS, xMSannotator, and mummichog [61,62,63]. It is expected that environmental chemistry knowledge will be incorporated into bioinformatic tools to facilitate the identification of xenobiotics.

**Figure 1:**
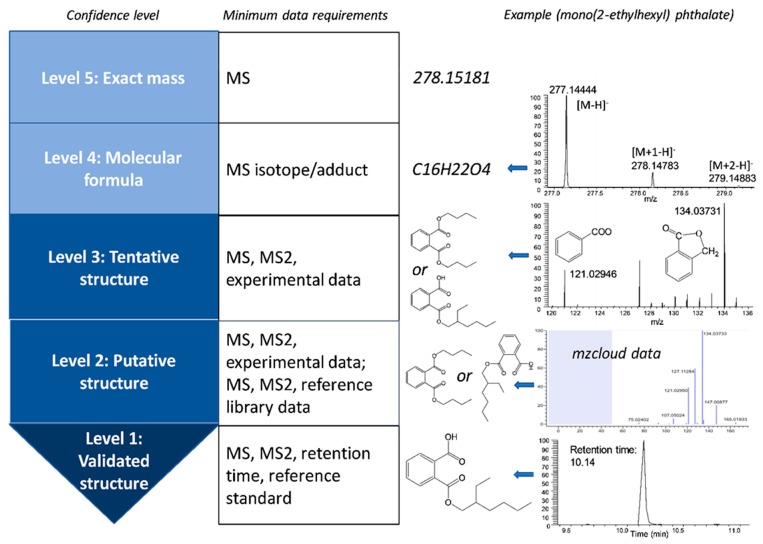
Proposed confidence levels in unknown xenobiotic identification with high resolution mass spectrometric analysis and exemplified with mono(2-ethylhexyl) phthalate.

The workflow mentioned above works well for identifying chemicals in one single sample. When the comparison across different samples is involved, other analyses are needed, such as peak aligning across samples, data pretreatment, and statistical analyses [25]. There are a wide range of chemometric and bioinformatic tools available at each stage in HRMS data analyses: preprocessing, annotation, and statistical analysis [52,64,65]. A variety of workflows, which encompass all stages of HRMS data analyses, are also available, including workflow4metabolomics, galaxy-M, XCMS online, MetaboAnalyst, MAVEN, MAIT, and MZmine 2 [64]. To standardize the confidence level in unknown identification, a variety of systems have been established to communicate the confidence [66,67]. In general, five levels are present and listed as follows with the increase of confidence, as shown in Figure 1: exact mass only, unequivocal molecular formula, multiple putative chemical structures available, one putative chemical structure, and validated structure with reference standard match in both retention time and mass spectra [68]. 

Until now, targeted analysis, suspect screening, and unknown screening based on TOF instruments (mainly QTOF) have been used on a widespread basis to measure human exposures to a broad range of chemicals, including both persistent (e.g., organochlorine pesticides, polychlorinated biphenyls, polybrominated diphenyl ethers) and non-persistent (e.g., drugs, pesticides, surfactants, personal care products) chemicals [25,69]. One study developed a LC-QTOF/MS based suspect screening technique with a library of collision-induced dissociation accurate mass spectra of more than 2500 toxic compounds, including illegal and therapeutic drugs, pesticides, and alkaloids [70]. A large number of studies also reported the application of FT-Orbitrap instruments in xenobiotic screening including drugs and pesticides in both human and environmental samples [71,72,73,74,75,76]. Most studies are using the three analytical approaches together to capture a wide range of chemicals [39,76].

One research area in which HRMS has gained widespread application is metabolomics. Metabolome refers to the sum of all low molecular weight metabolites present in a living system [77]. Non-targeted metabolomics based on HRMS can simultaneously detect the endo- and exogenous chemicals, directly linking exposure to internal dose, biological effects, and disease pathobiology, which is a critical component of the exposome [78]. Thus, metabolomics has become a critical platform in exposome research. 

### 2.2. Biological Approaches

Human exposure to xenobiotics can induce changes of the biological functions in many respects, such as gene transcription and protein synthesis. Instead of directly measuring the exposure itself, biological approaches measure these biological changes to understand the influences of exposure on human health. This approach is particularly helpful for those exposures which cannot be measured directly, such as reactive agents. For instance, reactive electrophiles, including reactive oxygen and nitrogen species, aldehydes, oxiranes, and quinones, can rapidly react with DNA and protein once absorbed into the organism [79]. Measuring adducts of these electrophiles with blood electrophiles such as hemoglobin (Hb) and human serum albumin (HAS) can help assess the human exposure to reactive electrophiles [79]. 

Analytical techniques used in biological approaches can be either instrument-based (e.g., mass spectrometry), or effect-based (e.g., bioassays), or the combination of both [80,81,82]. As the instrument-based techniques have been discussed earlier, here we focus on the effect-based and combined approaches. Advantages of effect-based approaches are listed as follows: (1) covers a wide spectrum of modes of action (MOA); (2) provides the opportunity to investigate the actual molecular targets; (3) allows prioritization of individual organisms for further investigation as well as chemical groups for identifying relevant mixture components; and (4) is time/cost effective and can be applied in high through-put screening scenarios [80,83,84]. One recent study screened and evaluated internal exposures of turtles to chemical mixtures based on an in vitro effect-based approach including a battery of sensitive bioassays with different modes of action, including aryl hydrocarbon receptor (AhR)-mediated xenobiotics (AhR-CAFLUX), NrF2-mediated oxidative stress (AREc32), NFκB mediated response to inflammation (NFκB-bla), estrogen binding (VM7Luc4E2), and baseline toxicity (Microtox) [80]. 

In most cases, routinely measured target compounds only partly account for the biological effects of concern. Thus, a hybrid approach, or effect-directed analysis (EDA), a combination of biotesting, fractionation procedures, and chemical analytical methods, is proposed to identify non-target compounds that cause biological toxicity in the sample [85]. General procedures of EDA are as follows: (1) extract the compounds of interest in an effective and non-selective way; (2) clean-up the extracted samples fraction; (3) conduct bioassay to select the interesting sample fraction; (4) fractionate the selected sample fraction to reduce the complexity; and (5) identify the suspected compounds with instrumental analysis methods. EDA has become a successful strategy in identifying biologically active compounds in environmental samples [82,86]. Although this approach has scarcely been applied in biological samples because of the difficulty in sample preparation, it is a promising approach in identifying the unknown chemical exposure leading to certain specific toxicity endpoint [87]. For instance, by using EDA approach, one study revealed the presence of new environmental contaminants, including di- and two monohydroxylated octachlorinated biphenyls (octaCBs) and linear and branched nonylphenol (NP), in polar bear serum with transthyretin-binding potency bioassay [23]. 

One well known example of the biological approaches in exposome research is omics profiling, which has found applications in large scale studies at population level [78]. High-dimensional analytical platforms were usually employed in omics profiling [78]. Besides metabolomics, omics profiling approaches also include genomics, epigenomics, transcriptomics, and proteomics [78]. Within the exposome framework, these approaches can provide a deep understanding at system biology-level of how chemical exposure influences the human health [78]. An unprecedented source of information has been produced with respect to the effective biological effects of exposures at omic-level. This omics data can be used in the generation of novel hypothesis to discover the disease etiologies of chemical exposure.

### 2.3. Other Approaches

Human exposome encompasses all types of exposures throughout the life course, including those from exo- and endogenous processes at individual level (e.g., environmental contaminants and infection) and general exposures at global level (e.g., climate and social economic status) [20,78]. Biological approaches can identify the influences of these exposures on human health, but are not able to characterize the exposure source, identify the route of exposure, and provide a picture of spatial and temporal variability of the exposure, which are critical in establishing links between exposome and biological significance [20,78]. While a few external exposures (e.g., environmental contaminants exposure) can be assessed through chemical approaches most external exposures (e.g., air quality and social economic status) need to be measured through other approaches such as questionnaires and static monitors [20,78]. Recently, a variety of novel assessment methods have been employed to quantify the external exposures, including those methods based on geographic information systems, environmental sensors, and personal sensing technology [20,78]. Although this review focuses on the analytical approaches employed in characterizing the chemical exposome, it is highly stressed that these new external exposure measurement methods are an integral part of the entire exposome measurement. With the rapid advancement of technologies, increasing popular applications of these novel methods are expected in exposome studies at both individual and population levels.

## 3. Measurement-Based Exposome Studies

Commonly-used strategies for chemical exposome study include top-down and bottom-up approaches [1]. A top-down exposome strategy starts with measuring all chemicals in a subject’s (cases and controls) biospecimen, such as blood, at each life stage either through direct measurement or by investigating the physiological effects of exposures. After identifying the variant with significance by a series of data analysis, biological annotation is followed to determine the biological significance of the critical variable. This approach covers both exogenous and endogenous chemicals in the internal chemical environment, offers an efficient means for profiling individual exposures, and is the predominant strategy used in chemical exposome studies to date. A bottom-up strategy starts with a complete measurement of all the chemicals present in each external source (e.g., air, food, water, etc.) of a subject’s exposome at each time point. After determining the analyte possessing significant association with health outcomes, uptake and metabolism of the analyte in the human body is evaluated. This approach requires enormous efforts in identifying the important chemicals in various environmental media and would miss important endogenous components in the body generated due to non-chemical factors such as physical activity, noise, inflammation, and social stress. One significant advantage of this approach is that it can provide valuable information regarding the critical chemicals present in each external source, making it complementary to top-down approach. Details of the two approaches as well as the available exposome studies based on these two approaches are discussed below.

### 3.1. Top-Down Exposome Approach

The research process of a chemical exposome study employing a top-down approach can be divided into five steps theoretically. (1) sample preparation, including both the collection of samples from groups of population (e.g., health and diseased) and the pretreatment of samples for instrumental analysis; (2) sample analysis, including the selection of instrumental types and methods; (3) data analysis, including chemometric analysis of the raw data and statistical analysis of the processed data; (4) biological annotation, determining the biological significance of the critical exposure identified; and (5) source identification, characterizing the source of the critical exposures identified. 

Biomonitoring of biospecimens such as blood has several advantages for the exposome research. Firstly, it allows for the simultaneous measurement of exposures and the metabolic phenotypes. Secondly, it allows for the development and measurement of biomarkers for historic and current exposures. Any exposure including both chemical and non-chemical factors can leave unique signatures in the human body, even in the case of an exposure that has passed a long time ago [88,89]. If the signature is persistent and irreversible, we can assess the health outcomes of historic exposure. Thirdly, it allows for the development and measurement of biomarkers for disease at any stages. There is a long way to go from exposure to observed effects: external dose; internal dose; target organ dose; target organ metabolism; target organ responses; cellular/subcellular dose and interaction; toxic response; observed effects [90]. The biomarker development of diseases can help explain the roles of environmental exposures in the pathogenesis of diseases as well as facilitate the diagnostic and prognostic of diseases. Lastly, it allows for the retrospective analysis of samples. Biospecimen samples can be stored for a long period of time in appropriate conditions. 

Many large cohort studies are using targeted approaches or the combination of both targeted and non-targeted approaches to collect a wide range of exposures, followed by statistical analyses to investigate the relationships between important groups of environmental exposure as well as the associations between exposure and disease. For instance, INfancia y Medio Ambiente (The INMA), a birth cohort study in Spain, investigated the levels of exposure to a wide array of pollutants during pregnancy such as brominated flame retardants, perfluoroalkyl substances, and metals, aiming to examine the role of environmental pollutants during pregnancy and early childhood in relation to child growth and development [91]. The existing exposome initiative in the U.S., the Children’s Health Exposure Analysis Resource (CHEAR), is using both targeted and non-targeted analyses through a network of laboratories to provide a comprehensive measurement of myriads of environmental xenobiotics and biological response indicators in various biological samples to better understand the roles of environmental factors in children’s health [7]. 

In addition to the national efforts towards exposome, many individual laboratories in the academic field are also dedicated to the exposome research. Dr. Dean Jones’ group established an automated workflow for non-targeted exposome analysis with a dual chromatography (DC)-FTMS, combining a reverse phase C18 chromatography and anion exchange (AE) chromatography [24]. An adaptive processing software package, apLCMS, was exclusively designed for LC-FTMS data analyses [24]. The addition of C18 column increased the *m*/*z* feature detection by 23–36%, yielding a total number of features up to 7000 for individual samples [24]. This exposome workflow was capable of detecting environmental chemicals in the nanomolar and sub-nanomolar concentration ranges [92]. It has been extensively used in multiple studies to simultaneously detect endogenous metabolites with plasticizers, insecticides, fungicides, herbicides, drugs, bacterial products, and correlate environmental chemical exposure with a variety of health outcomes, including tuberculosis disease and neurological development [93,94,95,96,97,98,99].

By combining redesigned METLIN Exposome database with XCMS platform and cognitive computing, a newly established nontargeted workflow allows the detection of endocrine disrupting chemicals at low-nanomolar concentrations in human serum and urine and also allows the readout of the biological effect of a chemical [100]. An innovation of this workflow is using artificial intelligence as a potential tool to prioritize findings in exposome studies [100]. It is expected that this workflow will be more extensively used in future exposome research.

### 3.2. Bottom-Up Exposome Approach

The complete research process of bottom-up approach-based exposome studies include the following procedures: (1) sample preparation, including sample collection from all the potential external exposure sources and sample pretreatment prior to instrumental analysis; (2) sample analysis, identifying the chemicals present in the samples; (3) data analysis to identify the compounds associated with the disease of interest; and (4) biological validation, studying the metabolism and toxicity of the selected analytes in animal or cell-based models to confirm the relationship hypothesized. 

This approach helps identify the critical exposure sources, therefore facilitating the authority or person to take actions to mitigate exposures. It is beneficial for studying those health outcomes which can be mainly ascribed to external exposome, such as allergies [101]. Although genetic factors also contribute to the incidences of allergies, it has been recognized that the increase in allergies observed in the past decades can be explained exclusively by environmental changes occurring in the same time period [101]. A number of studies have suggested that a variety of air pollutants, including volatile organic compounds, formaldehyde, toluene, and polycyclic aromatic hydrocarbons, not only exacerbate but also cause many types of allergies such as atopic dermatitis [102,103,104]. However, known environmental factors found with a traditional research paradigm cannot explain the increase in the prevalence of allergic diseases worldwide. Therefore, a recent study called for the integration of the external exposome in the etiopathogenesis of these diseases since a wide range of environmental factors were involved [101]. 

This approach can also be used to understand the metabolism of xenobiotics in biological organisms. By comparing the mass spectra arising from the environmental media and the organisms living in it, for instance, water and fish, it is possible to differentiate the xenobiotics, metabolized xenobiotics, and endogenous metabolites in the organism [105].

The major limitation of this approach is the laborious workload needed in acquiring the complete exposome from endless external sources. Therefore, careful selection of exposure sources is crucial in the successful identification of the critical exposure related with the target disease. Knowledge of potential mechanisms of disease and the differences between cases and controls are helpful in the selection of appropriate exposure sources. 

## 4. Publicly Accessible Data-Based Exposome Studies

The ultimate goal of exposome study is to investigate the role of exposome in the pathogeneses of human diseases. Because of the huge challenge in the measurement of exposome, a few studies focused on those human exposures with publicly-available data [14]. Although this data is neither comprehensive across all exposure domains nor longitudinal, it provides an important platform for generating and testing hypothesis between exposome and human health. Such platforms allow the simultaneous analysis of multiple types of exposure (e.g., chemical exposure, social economic status, etc.). One successful example is the National Health and Nutrition Examination Survey (NHANES), a biannual health survey conducted by the U.S. Centers for Disease Control and Prevention, which provides information about the range of representative exposures across the general population. The NHANES includes environmental exposures such as chemicals, nutrients, and infectious agents and the measurement tools include LC/GC-MS, immunological assays, and questionnaires. 

Using cross-sectional data from NHANES, Patel et al. employed an Environmental-Wide Association Study (EWAS) approach to investigate the relationships between 266 unique environmental factors and the clinical status for type 2 diabetes [106]. EWAS relies on linear regression models fitted independently for each covariate to separately examine the association between single exposure factor and the health outcome [106]. Then the factors with significant associations were validated across all models [106]. EWAS approach has been used on a widespread basis to assess the comprehensive relationships between a broad range of environmental/behavioral/clinical factors and various types of diseases, including blood pressure [107], type 2 diabetes in the Marshfield Personalized Medicine Research Project Biobank [108], all-cause mortality [109], and telomere length [110]. Besides EWAS, other approaches were also proposed to assess the effects of multiple chemical and non-chemical environmental stressors on health outcomes. For instance, by combining “big data”, computational tools, and traditional biostatistics, one research evaluated putative relationships between exposures from natural, built, and social environment domains and lung cancer mortality and mortality disparities across four race and gender groups [111]. A total of 2162 chemical and nonchemical environmental stressor was involved in the study [111]. 

To move from a single exposure-disease analysis paradigm to cumulative exposure-disease models, advanced biostatistics are required to tackle large, multiple, heterogeneous, and secondary datasets. One recent study compared the performance of commonly-used multiple linear regression statistical methods within exposome context (237 exposure covariates), including EWAS, EWAS-multiple linear regression (MLR), Elastic net, sparse partial least squares regression, Graphical Unit Evolutionary Stochastic Search, and Deletion-Substitution-Addition algorithm (DSA) [112]. Authors found that none of the statistical methods outperformed others across all scenarios and properties examined. However, overall, multivariate methods outperformed univariate approaches in investigating the exposome [112]. Barrera-Gomez et al. extended this work by considering scenarios with statistical interactions and by providing a systematic comparison of methods that have been recommended to search for interactions [113]. In this study, Group-Lasso INTERaction-NET (GLINTERNET) and DSA had better overall performance than the other methods in detecting two-way interactions, but the sensitivity and false discovery rate was compromised [113]. Therefore, none of the statistical methods has outperformed others in analyzing such a large number of exposures so far. Many factors can affect the selection of the right exposure variant, such as highly correlated exposures and multiplicity. Patel and Ioannidis argued that effects that survive multiplicity considerations and that are large may be prioritized for future scrutiny in the exposome studies [114]. Future efforts may focus on other statistical methodologies such as profile regression, cluster analysis, and even machine learning methods in tackling large exposome datasets [112]. 

Several metabolome databases also provide essential information about the exposures, such as levels of xenobiotics and their metabolites in the biospecimens. HMDB, currently one of the world’s most comprehensive metabolome database, is a great example [115]. Its latest version contains 114,100 metabolites and 21,834 xenobiotics and their metabolites [115]. Many studies have been conducted to interrelate the xenobiotics and their metabolites with endogenous metabolites, metabolic pathway, and health outcomes based on HMDB and other publicly available information. Rappaport et al. obtained human blood concentrations of 1561 small molecules and metals derived from foods, drugs, pollutants, and endogenous processes from the literature [compiled by the Human Metabolome Database (HMDB) and NHANES], and mapped chemical similarities after weighting by blood concentrations, disease-risk citations, and numbers of human metabolic pathways [22]. The results showed that endogenous chemicals, drugs, and food chemicals have similar concentration ranges in human blood, whereas those of pollutants were 1000 times lower [22]. While chemicals in the four classes were equally studied in terms of disease risks, studies of metabolic pathways were dominated by endogenous molecules and essential nutrients [22]. Bessonneau et al. obtained concentrations of 1233 chemicals that had been detected in saliva from the literature integrated into the HMDB, then connected salivary metabolites with human metabolic pathways and PubMed Medical Subject Headings (MeSH) terms, followed by pathway enrichment and pathway topology analyses [116]. The study found that 196 salivary metabolites with KEGG id were mapped into 49 metabolic pathways and associated with human metabolic diseases, central nervous system diseases, and neoplasms [116]. Saliva exposome represents at least 14 metabolic pathways, including amino acid metabolism, TCA cycle, gluconeogenesis, glutathione metabolism, pantothenate and CoA biosynthesis, and butanoate metabolism [116]. These studies offer insights into the roles of environmental factors in the etiology of diseases from a systematic perspective.

## 5. Challenges in Exposome Research

The introduction of exposome is expected to have breakthrough changes in uncovering the secrets of human diseases. However, a variety of challenges are present at each step of exposome research, measuring the exposome and linking it with health outcomes [17,117]. These challenges as well as the potential solutions are briefly discussed in this section with a focus on the chemical exposome research.

### 5.1. Challenges in Measuring the Exposome

The complexity and heterogeneity nature of exposome and its dynamic variation in both time and space presents a huge challenge in measuring the exposome [18]. The current most comprehensive approach is constructing epidemiological cohort studies with large sample size and long-term follow-ups. Human samples such as blood are collected at each critical stage, including fetal, early postnatal, childhood, teenage, and adult, from the same population. EXPOsOMICS and HELIX projects in the European Union and CHEAR project in the U.S. are exemplary studies regarding exposome research [7,118,119]. However, such studies are usually costly and laborious, and unaffordable for individual laboratories. There is an urgent need for alternative approaches to lower the cost of exposome research. Tooth and hair matrix biomarkers can incorporate the intensity and timing of exposure and has been referred to as “retrospective temporal exposome” [120,121,122]. This provides an effective approach to study the historical exposures in human beings for individual principal investigators.

Another challenge is that no single analytical technique can exhaust the chemical exposome in one sample because of the remarkable differences of chemicals in a wide range of physicochemical properties including mass, polarity, abundance, lipophilicity and pKa [89,123]. Even with the same technique, different sample processing methods and parameter settings can influence the results significantly. To measure as many chemicals as possible, samples should be carefully portioned and appropriately processed to fit different analytical techniques, which is costly and laborious.

Xenobiotics and their metabolites in the biological samples are usually at trace levels with several orders of magnitude lower than that of endogenous metabolites [22]. This demands high sensitivity for analytical instruments, which is also one of the reasons that mass spectrometry-based analytical platforms are gaining popularity in chemical exposome measurement. Low abundance mass spectra are significantly affected by the instrumental noise. Further, isotope pattern observed in high abundance mass spectra is usually not available for xenobiotics, which increases the difficulty in the identification of these compounds. Recently, efforts are being made to increase the intensity of these low abundance signals. For instance, one research group from Singapore isotopically labelled those xenobiotic biomarkers with common functional groups including phenolic hydroxy, carboxyl, and primary amine [124]. This method has improved sensitivity of 2–1184 fold for xenobiotics compared with other mass spectrometry based methods [124]. It has also been reported that increasing the number of replicate injections can help improve the reliability in low abundance chemicals measurement in high resolution metabolomics [125]. One recent study recommends integrating ion mobility spectrometry into mass spectrometry-based exposome measurements, which can provide increased overall measurement dynamic range and thus result in frequent detections of lower abundance molecules that are previously undetected [68].

HRMS data analysis such as unknown identification is also a huge challenge in HRMS based chemical exposome studies. None of the chemometric and bioinformatic tools available can successfully group and align all the features correctly. Every algorithm has its own pros and cons. In addition, although the compound databases are increasing the coverage annually, they are still far behind the number of chemicals available. More than 60 million chemicals are present in PubChem, however, only around 220,000 MS/MS spectra from 20,000 molecules or so are accessible in the databases [126]. 

### 5.2. Challenges in Associating Exposome with Diseases

In exposome, we are dealing with thousands of environmental risk factors that vary by source, place, and time. These factors affect human health differently depending on the exposure route, exposure timing window, dose, and specific target organ. In addition, these factors can also interact with each other to have synergistic, additive, or antagonistic effects when exerting effects on certain health outcome. To fully understand the effects of exposome on human health, it is necessary to integrate all the exposure factors and evaluate their effects systematically, which poses a great challenge. 

In addition, exposome mapping could discover hundreds or even thousands of altered molecular features associated with disease endpoints. However, it is difficult to identify key exposome features that may drive disease or contribute to disease etiology. To address this, advanced statistical methodologies, such as machine learning and artificial intelligence, hold the promise of pinning down molecular features that play a key role in the pathogenesis of human disease. 

## 6. Future Directions

Many factors can contribute to the further development of exposome research, including advancement in analytical platforms, high-throughput statistical analysis, HRMS data mining algorithms, large database of chemical substances and mass spectra, and biochemical pathways. It is the rapid advancement in the high-resolution metabolomics techniques that provides a high-throughput and affordable platform for the monitoring of environmental exposures in human beings. Instruments with high sensitivity, broad dynamic range, high resolution, high mass accuracy and a low cost are desirable in measuring the chemical exposome. Exciting achievements are also expected from the development of effective bioinformatic computation. Excellent algorithms are needed to help remove the noise in the mass spectra, group peaks, generate MS/MS spectra and retention time information, etc. It is time for exposome to embrace techniques such as machine learning.

With the establishment of exposome ontology through national studies such as CHEAR, more exposome researches are expected from the academic field. Thus, it is necessary to establish standards to allow cross comparison and validation. If every study follows the established standards, the data can be used for the integrative and systematic study in the future contributing to the complete picture of the exposome research. To establish the relationship between exposome and health outcomes, traditional statistical analysis needs to embrace big data analysis techniques to pinpoint the critical exposures. To better correlate the exposome with biological effects, high-throughput analytical techniques are needed to integrate data in exposome with other omics analyses including genome, transcriptome, proteome, and epigenome. This will help the principal investigators to study the mechanistic basis of the exposome in an affordable way. 

## 7. Conclusions

Exposome research paradigm provides a great opportunity to identify critical non-genetic factors that contribute to the onset and progress of various diseases. This paper discussed the commonly used measurement techniques in chemical exposome research and reviewed the available chemical exposome studies. As the technologies moving forward along with the establishment of exposome ontology, more exciting discoveries are waiting in the journey to uncover the roles of non-genetic factors in the pathogeneses of human diseases.
